# Cognitive Research and Mathematics Education—How Can Basic Research Reach the Classroom?

**DOI:** 10.3389/fpsyg.2020.00773

**Published:** 2020-04-23

**Authors:** Henrique Simplicio, Hedwig Gasteiger, Beatriz Vargas Dorneles, Ka Rene Grimes, Vitor Geraldi Haase, Carola Ruiz, Francéia Veiga Liedtke, Korbinian Moeller

**Affiliations:** ^1^Developmental Neuropsychology Laboratory, Biological Sciences Institute, Neurosciences Department, Universidade Federal de Minas Gerais, Belo Horizonte, Brazil; ^2^Mathematics Education, School for Mathematics and Computer Science, Institute of Mathematics, Osnabrück University, Osnabrueck, Germany; ^3^Post-Graduate Program of Education, School of Education, Universidade Federal Do Rio Grande Do Sul, Porto Alegre, Brazil; ^4^Department of Special Education, The University of Texas at Austin, Austin, TX, United States; ^5^Department of Psychology, Universidade Federal de Minas Gerais, Belo Horizonte, Brazil; ^6^Neurocognition Department, Universidad Católica del Uruguay, Montevideo, Uruguay; ^7^Post-Graduate Program of Psychology, Psychology Institute, Universidade Federal Do Rio Grande Do Sul, Porto Alegre, Brazil; ^8^Centre for Mathematical Cognition, Loughborough University, Loughborough, United Kingdom; ^9^Leibniz-Institut für Wissensmedien, Tübingen, Germany; ^10^Department of Psychology, University of Tübingen, Tübingen, Germany; ^11^LEAD Graduate School & Research Network, University of Tübingen, Tübingen, Germany

**Keywords:** numerical cognition, transfer of knowledge, applied science, math education, evidence-based practice, research-to-practice

## Introduction

Numeracy is critically associated with personal and vocational life-prospects (Evans et al., 2017; Grotlüschen et al., 2019); yet, many adults and children lack a basic level of proficiency (Jonas, 2018). At the same time, research interest in numerical cognition, and its neuro-cognitive foundations (e.g., Cohen Kadosh and Dowker, 2015), as well as in mathematics education (e.g., Dennis et al., 2016) continues to grow. In this opinion, we argue that more intensive discussion across the disciplines is necessary to answer the question how results from basic research can make it to the classroom, how classroom practices can be validated by research, and discuss a theoretical framework for guiding future transfer endeavors.

Transferring basic research results to educational praxis is not a new challenge. As early as 1899, James (1958) noted the difficulty of directly deriving suggestions for pedagogical practice from psychological research. Even when successful, research in psychology might not be enough to derive effective suggestions or direct conclusions for educational practice without considering environmental challenges and requirements of teaching. Clearly not all basic research aims at informing educational practice; however, failure of important results from research to successfully impact practice reflects missed opportunities at some point during dissemination—as is failing to validate effective existing practices through research to allow for what may be called *practice-based evidence*.

## Basic Research, Applied Research, and Use-Inspired Basic Research

To illustrate possible barriers for moving basic research results on numerical cognition into the classroom, Stokes' Quadrant Model of Scientific Research (Stokes, 1997) may be considered. Agnostic to a specific discipline, Stokes offered two dimensions to visualize goals of research: research inspired by the quest for fundamental understanding vs. research specifically designed with consideration of use. Stokes emphasized that the two dimensions do not describe two opposite poles on a linear scale because if so, the quest for fundamental understanding and consideration of use would drift apart, or at least would not be connected. Additionally, Stokes described a category syncing basic research with more applied research which he termed *use-inspired basic research*. Research in this category is inspired by the quest for fundamental understanding, with the idea to explicitly consider usefulness for practical needs.

Disciplinary fields such as the learning sciences, cognitive science, neuroscience, and educational psychology may overlap in terms of more basic or more applied research. For reasons of parsimony, we conceptualized *more basic research* as that conducted in the disciplines of neuroscience, cognitive science, biology, and genetics. In contrast, we conceptualized *more applied research* as research in the disciplines of mathematics education, educational psychology, and the learning sciences. Moreover, we operationalized *use-inspired basic research* as research conducted by any of the above disciplines explicitly for use in educational contexts. Of course, each of these disciplines operates on different levels of observation (e.g., brain, individual, classroom) and therefore contributes considerably to our understanding of numerical cognition from the neuro-cognitive foundations to the acquisition and teaching of numerical skills. In the following, we provide examples of research from several fields.

## More Basic and More Applied Research on Numerical Cognition

The number of meta-analyses published since 2015 manifests the contributions from both more basic and more applied research. Examples of more applied research on numerical cognition include evaluations of effectiveness of interventions in early childhood (Mononen et al., 2014; Wang et al., 2016; Christodoulou et al., 2017; Nelson and McMaster, 2019); for older students (Jitendra et al., 2018; Stevens et al., 2018); across age groups (Dennis et al., 2016); and across different regions of the world (Conn, 2017). Other examples include interventions for students with emotional difficulties (Losinski et al., 2019); math anxiety (Namkung et al., 2019); or on attitudes toward achievement (Savelsbergh et al., 2016); the impact of homework (Fan et al., 2017); and specific teaching strategies (Capar and Tarim, 2015; Rittle-Johnson et al., 2017; Guillaume and Van Rinsveld, 2018).

On the other hand, meta-analyses of more basic research include synthesized results on the association of numerical and spatial cognition (Hawes et al., 2019); magnitude understanding (Vanbinst and De Smedt, 2016; Sokolowski et al., 2017); rapid automatized naming (Koponen et al., 2017); specific brain regions associated with numerical cognition (Yeo et al., 2017); specific numerical processes (Arsalidou et al., 2018); specific cognitive functions (Peng et al., 2016); different numerical representations (Schneider et al., 2017); and genetic influences (Chen et al., 2017; King et al., 2019).

The above list is far from exhaustive. Synthesizing the entire corpus of work-to-date to create a holistic understanding of what we currently do and do not know on numerical cognition, and then disseminating that work across disciplines and to educators, is a substantial challenge for moving research results into the classroom. Looking at just 15 evidence-based instructional practices, using three different procedures for either early or late implementation, Koedinger et al. (2013) explained that an educator would have to consider 205 trillion options; *and* the effectiveness of these instructional practices is susceptible to contextual variables (e.g., Dunlosky et al., 2013; Davenport et al., 2019).

## Research and Noisy Application in Classrooms

**Figure 1:**
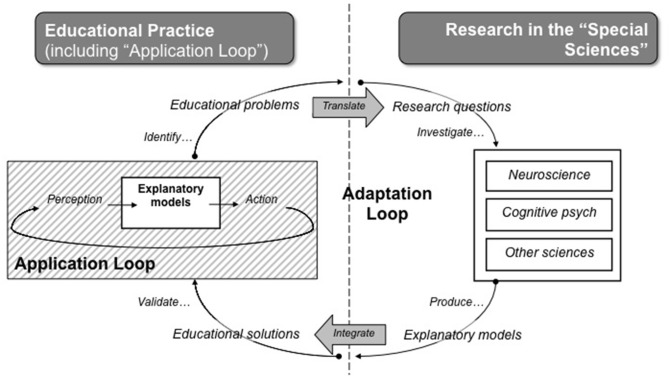
Adaptation Loop (Source: Connell et al., 2012, Reproduced with permission of Michael W. Connell ©2010–2014. All rights reserved. Author contact: Michael.W.Connell@gmail.com).

But how can research then come to influence classroom practice? And how can classroom practice influence what is researched? In our opinion, suggestions for two-way bridges over research-to-practice gaps (e.g., Bowers, 2016; Reynvoet et al., 2016; Mackey, 2019; Thomas, 2019) require more in-depth analysis. Where (Stokes, 1997) provides a macro-view, Connell's Adaptation Loop (2012, see Figure 1) provides a closer look.

The right chart of Figure 1 reflects research, whereas the left represents educational practice. Moving in clockwise direction, starting at the top left corner of the diagram, the process of research and adaptation illustrates recognizing a problem, translating the problem into research questions, investigating questions by scientific domain, providing explanations, designing solutions, validating solutions in the educational environment, and then repeating the process.

However, within the domain of educational practice, the *application loop* indicates the iterative nature of changes *within* educational settings and reflects the necessity for further adaptation during the validation process. We suggest the *Application Loop* model as an accurate reflection of what occurs within education. Education is not a unitary system, but a system made up of different sub-systems with hierarchies of stakeholders (i.e., policymakers, administrators, teachers, students). Implementation of explanatory models or interventions previously proven effective in basic research often fails to produce similar results in educational practice because, at each hierarchical level, humans make decisions which introduce new variables. While researchers are cognizant of some of these variables, and often consider these as noise with the aim to control these through experimental design or statistical models, this noise may be the key to the comprehension of use-inspired basic research.

### Listen to the Noise

Collaboration, whether across disciplines or within educational contexts, with the explicit aim of conducting use-inspired research, is not easy. Berliner claimed that education research “is the hardest science of all” (Berliner, 2002, p. 18). Below we discuss a few issues critical for researchers to consider when planning to conduct use-inspired research.

First, research in the classroom interrupts daily business of teachers and students. Moreover, testing in classrooms and controlled interventions change the typical dynamic of teaching and learning. Evaluating the effectiveness of interventions may necessitate students' absence from the classroom. Such interruptions not only let students miss instruction but may also disturb learning progress of other students. Students may either come to resent being pulled from their classroom or resent not being pulled when not assigned to the treatment group. These circumstances reflect conflicts of goals between the parties involved in use-inspired research, which may lead to tensions.

Additionally, researchers are interested in publishing their work, thus strive for theoretically and methodologically sound but also positive results. Therefore, they have to include multiple and different measures to evaluate effects of interest, or to control for potential moderators, mediators, or confounds. However, time in classrooms is limited and a precious resource. Schools have demands, schedules, and goals, which are different from those of researchers. This discrepancy often leads to a zero-sum game, in which compromises to meet the needs and interests of both schools and researchers may impact outcomes. Careful consideration of the cost/benefit of variables likely to inform research results requires balancing the cost to the students/teachers/schools and the benefits to science.

### How to Increase Use-Inspired Basic Research

There have been others advocating for use-inspired basic research with careful consideration of how to increase implementation and ecological validity of research (e.g., Cai et al., 2017, 2018, 2019). For example, Smolkowski et al. (2019) provided suggestions on levels of implementation, and Higgins et al. (2019) focused on how research can become more use-inspired:

- Choose outcome measures that matter to educators in their context
- Include educators and students in the research process (i.e., researching with them not on them)
- Be flexible and sensitive to time and schedules
- Consider that research that was effective in the lab may not be effective in the classroom
- Ask questions educators want and need to have answered
- Disseminate findings in non-academic media (i.e., social media, websites); attend educator specific conferences.

Space limitations do not allow us to provide multiple successful examples of such use-inspired research (e.g., Hawes et al., 2020), of research partnerships (e.g., Kaplan et al., 2019), or of societies actively promoting and including educators during their annual conferences (e.g., The Math Cognition Learning Society, The International Mind, Brain, and Education Society, The Earli SIG 22). We recommend readers consider the above citations as references for how to reframe perspectives of what it means to conduct use-inspired research. Additionally, researchers interested in what teachers are doing in the (math) classroom can follow the Twitter hashtags #mtbos, #iteachmath, and #SwDMathChat. These clearly indicate that educators often ask the same questions as researchers; although usually without the benefit of being able to validate their work beyond their personal and peer experiences. Collaborative work is happening, though not yet at scale. For example, educator Simon Gregg and researcher Tali Leibovich-Raveh co-authored a paper on numerical magnitude understanding after several discussions on Twitter (preprint: https://osf.io/ndyb6/). Sharing preprints via social media, talking to educators face-to-face, going to educator focused conferences, or any other means of closing feedback loops are examples of ways to move research on numerical cognition forward within and across disciplines.

## Interdisciplinary, Collaborative Research: A Way to Bridge the Gap?

More than 100 years ago, James (1958) not only described the difficulty of directly deriving suggestions from psychological research to pedagogical practice; he also claimed that research must include the expertise of educators to respect the complexity of teaching in classrooms. A first step would be when basic and more applied research on numerical cognition find a shared vocabulary and bring their expertise together to do interdisciplinary use-inspired basic research (i.e., Stokes, 1997). Moreover, going from the lab to the classroom and vice versa could offer new perspectives for teaching and learning. Connell et al. (2012) idea of application loops points to the next steps by indicating the necessity of iterations at the application stage to consider contextual demands of classroom practice. To illustrate, imagine various entities in Connell et al. (2012) as overlapping concentric circles in a Venn Diagram: circles for each domain of applied and basic research, and also circles for the different stakeholders in educational practice. Maybe, any two circles will overlap, or some may overlap with more than one other circle, but in the best case, all circles should overlap at a shared core. Each circle is necessary, but the point at which all circles overlap is where use-inspired, contextually relevant research occurs. There will always be a need for basic research, which may not directly impact use, and many open questions remain for researchers to explore. In contrast, classroom teachers have context-specific and practice-relevant questions for research. We propose that results from research should find their way into classrooms, but we need more integration of different perspectives and fruitful collaborations between researchers of different disciplines with educators. Only then we may have a chance to bring results from basic research into educational practice. However, as Minshall (2009) put it, “knowledge transfer is a ‘contact sport'; it works best when people meet to exchange ideas, … and spot new opportunities.”

## Author Contributions

Each of the authors contributed equally to the theoretical framing of this article and contributed to edits throughout the writing process. HS wrote the initial draft based on notes from members of our workshop held at the Brazilian-German Numerical Cognition Winter School, Belo Horizonte, Brazil, August 2019. To acknowledge his work in transcribing our workshop notes, HS was considered the first author. HG located the call for manuscripts at Frontiers, managed the contributions of co-authors and finalized the manuscript. Therefore, we listed her as the second author.

## Conflict of Interest

The authors declare that the research was conducted in the absence of any commercial or financial relationships that could be construed as a potential conflict of interest.
